# Prevalence and Determinants of Household Self-Reported Diabetes Mellitus in Gauteng, South Africa

**DOI:** 10.3390/ijerph21111537

**Published:** 2024-11-20

**Authors:** Shoeshoe Mokhele, Tholang Mokhele

**Affiliations:** 1 Department of Pharmaceutical Sciences, School of Pharmacy, Sefako Makgatho Health Sciences University, Pretoria 0204, South Africa; 2 Geospatial Analytics, eResearch Knowledge Centre, Human Sciences Research Council, Pretoria 0002, South Africa

**Keywords:** diabetes mellitus, Gauteng, South Africa

## Abstract

Diabetes mellitus is one of the leading causes of morbidity and mortality worldwide. Type 2 diabetes mellitus is the most prevalent type of diabetes mellitus, and it is associated with both hereditary and lifestyle risk factors. South Africa is not exempt from this pandemic; hence, this paper aims to assess the prevalence and determinants of household self-reported diabetes mellitus in Gauteng, South Africa. Data were sourced from the Gauteng City-Region Observatory (GCRO) quality of life survey (2020/2021). Bivariate and multivariate logistic regressions were applied. The prevalence of household self-reported diabetes mellitus in Gauteng was 11.1%. The ‘other population’ group (which included Whites, Coloureds and Indians), as well as older respondents, higher household monthly food expenditure, poor self-perceived health status and household self-reported hypertension were factors that increased the odds of household self-reported diabetes mellitus. Only informal housing decreased the odds of household self-reported diabetes mellitus. Screening of diabetes mellitus among those with poor living conditions, no medical aid and lack of access to healthcare facilities such as Gauteng township and informal settlement residents should be intensified. This secondary disease prevention intervention is crucial, as it will enhance the appropriate referrals and timeous chronic treatment for those with diabetes mellitus.

## 1. Introduction

Diabetes mellitus is a chronic metabolic disorder characterised by hyperglycaemia, which is caused by a lack of insulin, insulin insufficiency and/or insulin resistance [1]. It is mainly classified as type 1 or type 2 diabetes mellitus. There is a lack of insulin in type 1 diabetes mellitus, while in type 2 there is insufficient insulin, insulin resistance or both. These result in decreased utilisation of glucose by body cells, leading to an increase in circulating glucose (hyperglycaemia). People living with diabetes would, therefore, experience fatigue and polyphagia as the cells are not able to utilise circulating glucose. The patients also become thirsty frequently, resulting in an increased intake of water and an increased rate of urination (polyuria) [2]. The glucose is excreted in the urine (glucosuria). The mentioned symptoms are experienced by both type 1 and type 2 diabetes patients. Type 1 diabetes mellitus develops at the childhood stage, while type 2 usually arises as one ages or in adulthood [3]. Diabetes mellitus may lead to several complications, such as neuropathy, retinopathy, nephropathy and cardiovascular diseases, such as heart disease and stroke [4]. Hence, diabetes is one of the major causes of increased morbidity and mortality associated with non-communicable diseases worldwide. In 2021, it was reported that 529 million people were living with diabetes mellitus globally, and it is estimated that the number of people living with diabetes will increase to 1.3 billion people by the year 2050 [3]. South Africa is not spared from this pandemic as it was estimated that about 4.6 million people aged between 20 and 79 years old were living with diabetes mellitus in South Africa [5]. The prevalence of diabetes mellitus has rapidly increased from 4.5% in 2010 to 12.7% in 2019 in South Africa [5,6]. Moreover, there is still a large proportion of South Africans living with undiagnosed diabetes mellitus [5,7,8]. Based on South Africa Demographic and Health Survey (SADHS) 2016 data, out of 6442 individuals, 67% were found pre-diabetic and 22% diabetic based on HbA1c test results [8]. Only 11% of the sampled population showed normal values of HbA1c. Diabetes mellitus was reported as the second leading cause of death in South Africa for the years 2015–2016 [9].

Of all diabetes mellitus cases, type 2 diabetes mellitus is the most prevalent type, constituting 96% of diabetes cases in adults [3]. Type 2 diabetes mellitus is associated with both hereditary and lifestyle factors [10]. The individual’s lifestyle is highly dependent on their social and financial status, as these may affect their diet selection as well as their physical activity/inactivity. Diet determines the calorie intake and plays a major role in the development of type 2 diabetes as it may lead to obesity, the major risk factor for type 2 diabetes [10,11,12,13]. An increase in urbanisation and busy life schedules in South Africa have given rise to the consumption of fast foods, highly processed foods and food with a high glycaemic index [14]. High intake of these foods increases the risk of obesity, insulin resistance and type 2 diabetes mellitus [15,16]. Though there are several factors that influence household choices of food, the consumption of certain food types may depend on affordability, hence the financial status of an individual or the household. Hypertension had also been found to be associated with diabetes mellitus in other previous studies [8,17]. In addition to diet and hypertension, a sedentary lifestyle is another contributor to the development of type 2 diabetes mellitus. Exercising or physical activity reduces the risk of obesity and the development of type 2 diabetes mellitus, and this is regarded as one of the non-pharmacological treatments for type 2 diabetes mellitus [18]. Other than both hereditary and lifestyle factors, sociodemographic factors such as population group, sex, age, marital status, level of education, income, occupation, socioeconomic status, social position and residential area have also been found to be associated with the risk of developing type 2 diabetes mellitus in South Africa [6,8,17,19,20].

Epidemiological studies that show the prevalence of diabetes mellitus in South Africa are very scarce. For instance, Pheiffer et al. [14] conducted a systematic review and meta-analysis of 1752 records, which were further reduced to 56 based on titles and abstracts eligibility, and finally, 11 studies met the inclusion criteria and were included in the systematic review. Of the 11 studies that were included, only two were nationally representative studies, the South African National Health and Nutrition Examination Survey (SANHANES) 2012 and the South African Demographic Health Survey (SADHS) 2016, whereas nine were community-based studies [14]. None of the nine community-based studies were focused on Gauteng province. The pooled prevalence in individuals 25 years or older was 15.25% [11.07–19.95%] for type 2 diabetes mellitus and 8.29% [4.97–12.34%] for newly diagnosed type 2 diabetes mellitus [14].

Gauteng is the smallest province in terms of area, but it is the most populated province, with a total population of 15,099,422 [21] (Stats SA, 2023). Males constitute 50.5%, while their female counterparts account for 49.5%. The province is comprised mainly of young people aged 15 to 34 years old, with 37.0% of the population, followed by those aged 35 to 59 years old with 31.9%. The majority of (88.5%) people live in formal housing or dwellings, followed by the population that lives in informal dwellings with 11.0% [21]. The prevalence of diabetes mellitus in Gauteng based on a HbA1c examination in 2012 was 7.9% [5.4–11.4] [16]. Interestingly, the self-reported rate of family history of diabetes in Gauteng in 2012 was 19.4% [17]. The prevalence of diabetes mellitus in women and men in Gauteng in 2016 was 9.3% and 6.6%, respectively [22]. In the absence of the latest statistics on the prevalence of diabetes mellitus, population-based surveys with self-reported diabetes mellitus can be used to provide such statistics. The validity of self-reported diabetes mellitus has been tested elsewhere using measured diabetes mellitus and medical records [23,24]. For instance, Okura et al. [23] found that the correlation between self-reported diabetes mellitus, hypertension, and stroke and medical records was substantial. Moradinazar et al. [24] reported the overall acceptable validity of self-reported diabetes mellitus in the study population with a correction factor of 25.3%. In both cases, the validity was associated with some sociodemographic factors such as age, sex and a family history of diabetes mellitus [23,24]. Furthermore, Ho et al. [25] assessed the relation of five self-reported chronic cardiovascular diseases and diabetes mellitus to self-perceived health with moderating effects of sex and age in Hong Kong, China. Therefore, the prevalence of self-reported diabetes mellitus in Gauteng, as well as the factors associated with it from the current study, are of great importance for provincial healthcare planning, as these provide the latest statistics on diabetes mellitus. Hence, the aim of this paper is to assess the prevalence and determinants of household self-reported diabetes mellitus in Gauteng, South Africa.

## 2. Materials and Methods

### 2.1. Data

Data utilised in this paper were obtained from the Gauteng City-Region Observatory (GCRO) quality of life survey 6 (2020/2021). This survey was conducted from October 2020 to May 2021. The GCRO quality of life survey is undertaken every two years. The survey is designed to be representative at a ward administrative level. Wards are subdivisions of municipalities used for political administration and election processes [26]. Multistage stratified cluster random sampling was used. The first stage entailed random probability proportional to size (PPS) sampling for the selection of enumerator areas (EAs) within each ward to serve as clusters for visiting points or households. The second stage involved a simple random selection of residential dwelling units as visiting points or households [26]. The target population was adults aged 18 years and older. Households headed by those aged 17 years old or younger were excluded from the survey. Data were collected using electronic tablets by trained fieldworkers who followed COVID-19 protocols [26]. The data were benchmarked using the GeoTerraImage (GTI) 2021 population estimates, which were based on the 2020 mid-year population estimates of Statistics South Africa (Stats SA) for the generalisability of the study findings to Gauteng’s adult population [26]. More details on the sampling design, data collection and benchmarking have been described elsewhere [26,27,28].

### 2.2. Measures

The primary outcome variable, household self-reported diabetes mellitus, was based on the following question, “In the last year, have you or any other member of this household had any of the following conditions? Diabetes mellitus”. The response was coded 0 = no and 1 = yes.

The explanatory variables included sociodemographic variables and other variables that were related to diabetes mellitus based on previous studies [6,8,17,19,20]. The sociodemographic factors included sex (male or female), population group (Black African or other), age (18–24, 25–34, 35–44, 45–54, 55–64 and 65+), education (secondary or less, matric and tertiary), household income in South African Rands (R), which is the total money brought to the household per month by all household members (R800 or less, R801–R3200, R3201–R12800 and R12801 or more), medical aid (yes or no), living standard satisfaction (satisfied, neither satisfied nor dissatisfied and dissatisfied), food satisfaction (satisfied, neither satisfied nor dissatisfied and dissatisfied) and household food expenditure in South African Rands (R) per month (R1000 or less, R1001–R2000 and R2001 or more). Other variables included perceived health status (excellent, good and poor), household self-reported hypertension (yes or no), healthcare facilities access (yes or no), healthcare facility type (private or public), health services satisfaction (satisfied, neither satisfied nor dissatisfied and dissatisfied) and dwelling type (formal or informal/other).

### 2.3. Data Analysis

Data analysis was performed using Stata version 15.0 [29]. The Stata “svy” command was considered to incorporate benchmarking weights during the data analysis for the generatability of the findings. Differences in the categorical variables were compared using 95% confidence intervals (CIs) and Chi-square tests. Bivariate logistic regression and multivariate logistic regression analyses were performed to assess the factors affecting self-reported diabetes mellitus within households in Gauteng, South Africa. All statistically significant variables, except household income and health facility type, from the bivariate logistic regression models were fitted into the final multivariate logistic regression model. Both household income and healthcare facility type were excluded in the final multivariate logistic regression model due to low responses for these variables, which could have resulted in a reduction in the sample size for the final multivariate regression model. In addition, they were both not statistically significant when included in the final multivariate logistic regression model. Adjusted odds ratios (aOR) with 95% confidence intervals (CIs) were reported from the final model. For all analyses in this study, a *p* ≤ 0.05 was considered for the level of statistical significance.

## 3. Results

### 3.1. Characteristics of Respondents

This study sample consisted of 13,616 respondents (Table 1). Female respondents accounted for 50.1%, and Black Africans constituted 90.4%. The majority of respondents were aged 25–34 years old (23.6%), followed by those aged 35–44 years old with 22.6%. The majority of participants had secondary or less education with 43.0% and fell under the R801–R3200 household income band with 37.2%. Most people (76.8%) did not have medical aid and were using public health facilities (74.7%). The majority of residents (65.8%) were satisfied with their healthcare services and lived in formal housing with 85.6%.

### 3.2. Prevalence of Household Self-Reported Diabetes Mellitus

Overall, 11.1% (95% CI [10.4–11.9]) of the respondents reported that they or any member of their household had diabetes mellitus in the last year preceding the study in Gauteng (Table 2). Self-reported diabetes mellitus within households was significantly higher among the ‘other population’ group (19.2%, 95% CI [16.9–21.6]), those aged 65 and older (23.6%, [20.8–26.7]), those who had household income of R12801 or more (12.9%, [10.9–15.3]), those who had medical aid (13.7%, [12.2–15.4]), those who were satisfied with their standard of living (12.5%, [11.5–13.6]), those who had household food expenditure of R2001 or more (13.9%, [12.3–15.7]), those who perceived their health status was poor (22.7%, [20.0–25.7]), those who reported that they or any of their household members had hypertension (27.7%, [25.7–29.8]), those who were using private healthcare facilities (13.6%, [12.2–15.3]) and those who were living in formal dwelling types (12.2%, [11.4–13.0]) compared to their respective counterparts (*p* ≤ 0.001 in all cases).

**Figure 1:**
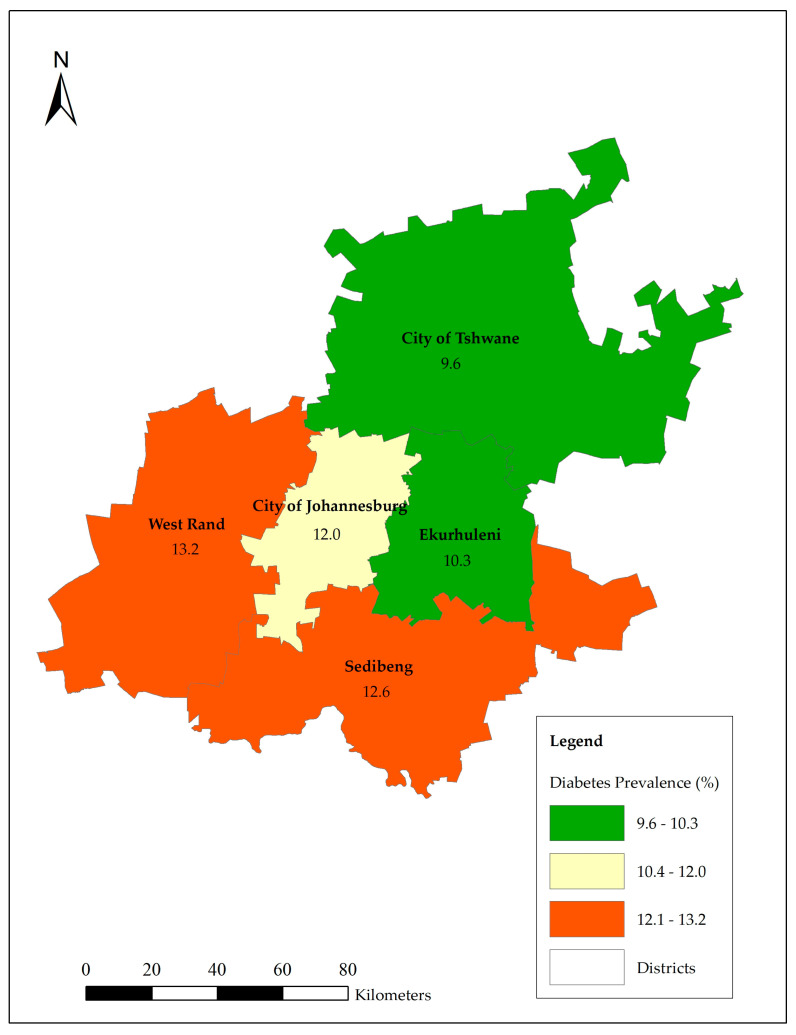
Prevalence of household self-reported diabetes mellitus by districts.

Figure 1 shows that West Rand had the highest prevalence of household self-reported diabetes mellitus with 13.2% followed by Sedibeng with 12.6%. The City of Tshwane had the lowest prevalence of (9.6%) household self-reported diabetes mellitus within households in Gauteng.

### 3.3. Multivariate Logistic Regression Model of Factors Associated with Household Self-Reported Diabetes Mellitus

Table 3 highlights a multivariate logistic regression model of factors associated with household self-reported diabetes mellitus within households in Gauteng, South Africa. The ‘other population’ group (which included Whites, Coloureds and Indians/Asians) was significantly (aOR = 1.70, 95% CI [1.33–2.18], *p* < 0.001) more likely to report that they or any member of their household had diabetes mellitus in the last year preceding the study compared to Black Africans. Older respondents were significantly (45–54 years old: aOR = 1.74, 95% CI [1.25–2.42], *p* = 0.001; 45–54 years old: aOR = 2.38, 95% CI [1.77–3.20], *p* < 0.001 and 65 years and older: aOR = 2.16, 95% CI [1.57–2.96], *p* < 0.001) more likely to report that they or any member of their household had diabetes compared to their younger counterparts aged 18–24 years old. Respondents who had a higher household monthly food expenditure were significantly (R1001–R2000: aOR = 1.44, 95% CI [1.18–1.74], *p* < 0.001 and R2001 or more: aOR = 1.41, 95% CI [1.11–1.80], *p* = 0.006) more likely to report that they or any member of their household had diabetes mellitus compared to those who spend R1000 or less on food per month. Respondents who perceived that their health status in the last four weeks preceding the survey was poor were significantly (aOR = 2.13, 95% CI [1.65–2.75], *p* < 0.001) more likely to indicate that they or any member of their household had diabetes mellitus than those who perceived that their health status was excellent. Respondents who reported that they or any household member had hypertension were significantly (aOR = 4.26, 95% CI [3.58–5.08], *p* < 0.001) more likely to report that they or any member of their household had diabetes mellitus than those who reported that there was no one with hypertension in their households. Participants who lived in informal houses were significantly (aOR = 0.51, 95% CI [0.37–0.69], *p* < 0.001) less likely to report that they or any member of their household had diabetes mellitus compared to those who lived in formal houses.

## 4. Discussion

From this study, the prevalence of household self-reported diabetes mellitus in Gauteng province of South Africa from 2021–2022 was 11.1%. This is higher than the prevalence of measured diabetes mellitus in Gauteng in 2012, which was 7.9% (95% CI [5.4–11.4]) [17]. However, it is worth noting that this prevalence of household self-reported diabetes mellitus falls within the 95% CI of the 2012 measured prevalence of diabetes mellitus. In addition, this prevalence of household self-reported diabetes mellitus in Gauteng is in line with the national prevalence of diabetes mellitus of between 9.5% and 12.7%, as reported elsewhere [5,6,17,19]. For instance, the prevalence of diabetes mellitus in South Africa was 11%, and the prevalence of self-reported diabetes mellitus was 6.86% [19]. The higher prevalence of diabetes mellitus in South Africa had been reported before as 18.6% and 22.0% [8,20].

With regard to the factors associated with household self-reported diabetes mellitus, the ‘other population’ group (which included Whites, Coloureds and Indians/Asians) had an increased odds of reporting that they or any member of their household had diabetes in the last year preceding the study compared to Black Africans. Similar findings were also reported by other studies in South Africa [6,17,20]. For instance, Sifunda et al. [6] found that the prevalence of diabetes mellitus was significantly higher among Indians, followed by White and Coloured people, compared to the Black African population.

This study highlighted that the odds of household self-reported diabetes mellitus increased with age. Previous studies have also found similar trends where diabetes mellitus increased with age [6,17,20,30,31]. These trends were also noticed even when sex stratification was considered; thus, for both males and females explored independently, diabetes mellitus increased with the age of the respondents [20].

Residents who had a higher household monthly food expenditure had higher odds of household self-report diabetes mellitus compared to those who spent R1000 or less on food per month. Although the type of food bought was not explored in this study, an unhealthy diet is one of the major risk factors for hypertension and diabetes mellitus [10,17]. In addition, Gauteng province is more urbanised than any of the other provinces in South Africa, and as alluded to by Pheiffer et al. [14], increased urbanisation and busy life schedules have resulted in the consumption of fast and highly processed foods in South Africa. Kristo et al. [32] found that diabetes mellitus was associated with emotional eating. Furthermore, an unhealthy diet can further modulate the positive or negative effects of other lifestyle choices for the individual diagnosed with diabetes mellitus [32].

Respondents who perceived that their health status in the last four weeks preceding the survey was poor had increased odds of household self-reported diabetes compared to their counterparts. Similarly, Grundlingh et al. [8] also found that there was a higher prevalence of diabetes mellitus among those who perceived their health status as poor at 27.2% compared to those who perceived their health status as good at 20.0%. Furthermore, Ho et al. [25] reported that there was a significant association between diabetes mellitus and poor self-perceived health, and this association was stronger in men and younger adults. Self-reported diabetes mellitus is highly dependent on a previous diabetic diagnosis; therefore, it is not surprising that those who were likely to have been diagnosed with diabetes mellitus perceived that their health status was poor in the last four weeks preceding the survey.

Unsurprisingly, household self-reported diabetes mellitus was related to household self-reported hypertension in this study. Diabetes and hypertension have been found to be related in other previous studies in both developing and developed countries [8,17,31,33]. Tsimihodimos et al. [31] further asserted that the development of hypertension and diabetes mellitus track or predict each other over time, while Zwane et al. [33] reported that hypertension (24%) was a common comorbidity among diabetes mellitus patients. Grundlingh et al. [8] also found that people who were taking medication for high blood pressure were 1.5 times more likely to have diabetes than those who were not taking such medication.

Participants who lived in informal houses had decreased odds of reporting that they or any member of their household had diabetes compared to those who lived in formal houses. This finding is supported by findings by Shisana et al. [17], who found that urban informal residents had the lowest prevalence of diabetes mellitus at 4.6%, while it was 11.3% among urban formal residents. Furthermore, the findings from this study showed that those who had a household income of R12801 or more, those who had medical aid, those who were satisfied with their standard of living and those who were using private healthcare facilities reported a higher prevalence of household self-reported diabetes mellitus compared to their counterparts; however, these factors were not significant in the final multivariate logistic regression model. This shows that those who are better off are more likely to know their diabetes mellitus status due to access to physical health examinations compared to those who are not well off. Furthermore, high rates of undiagnosed diabetes have been largely attributed to a lack of access to healthcare and poor healthcare systems [34]. Mutyambizi et al. [19] also found that the prevalence of self-reported diabetes mellitus in South Africa was pro-rich. In addition, diabetes mellitus was reported to be more prevalent among higher socioeconomic groups than lower socioeconomic groups, although this was becoming less so over time [35]. Furthermore, Madela et al. [36] indicated that Black African populations living in more deprived wards in KwaZulu-Natal had a lower prevalence of diabetes mellitus.

This study has some strengths and limitations. Regarding strengths, for instance, data were benchmarked for the generalisability of the findings to the population of Gauteng province. To the best of our knowledge, this study is among the pioneers of household self-reported diabetes mellitus in Gauteng province. This study further showed that household self-reported diabetes mellitus can be used to acquire an overall diabetes mellitus prevalence in conditions where a physical examination is not viable due to financial and human resource constraints. In addition, self-reported health or self-perceived health has been widely used and validated as an acceptable measure of health globally [37,38,39,40,41,42]. Furthermore, as GCRO surveys are undertaken every two years, the trends of self-reported diabetes mellitus in Gauteng can be explored over time and space. Amongst the limitations was household self-reported diabetes mellitus; therefore, it has the potential for social desirability bias. In addition, the primary outcome variable of household self-reported diabetes mellitus was based on the following question, “In the last year, have you or any other member of this household had any of the following conditions? Diabetes”. This means that there could be an under- or overestimation of diabetes mellitus, as this depended on the respondent’s recall memory or knowledge of any other member of the household who had diabetes. The way the question was presented poses some challenges for assessing the prevalence of self-reported diabetes. For a more accurate or better understanding of the prevalence and determinants of self-reported diabetes mellitus in Gauteng, future GCRO surveys should consider adding the following question: “In the last year, have you had any of the following conditions? Diabetes” in addition to the current one “In the last year, have you or any other member of this household had any of the following conditions? Diabetes”; or consider replacing the latter one with the former. Regardless of the above-mentioned limitations, the findings from this study contribute to our knowledge about diabetes mellitus in Gauteng, South Africa.

## 5. Conclusions

Overall, the prevalence of household self-reported diabetes mellitus in Gauteng was 11.1%. Determinants of household self-reported diabetes mellitus with higher or increased odds included the population group, age, food expenditure, self-perceived health status and household self-reported hypertension. Only informal housing decreased the odds of household self-reported diabetes mellitus in Gauteng, South Africa. The screening of diabetes mellitus among those with poor living conditions, no medical aids and a lack of access to healthcare facilities, such as Gauteng township and informal settlement residents, should be intensified. This secondary disease prevention intervention is crucial, as it will allow for the appropriate referrals and timely chronic treatment for those with diabetes mellitus. Diabetes mellitus surveillance across the country should also be considered for tracking and monitoring diabetes mellitus prevalence. Future research should explore the spatio-temporal analysis of self-reported diabetes mellitus in Gauteng as well as across the country.

## Figures and Tables

**Table 1:** Characteristics of respondents.

	Sample	%	95% CI
Total	13,616	100.0	
Sex			
Male	6340	49.9	[48.8–51.0]
Female	7276	50.1	[49.0–51.2]
Total	13,616	100.0	
Population group			
Black African	10,933	80.4	[79.5–81.3]
Other	2683	19.6	[18.7–20.5]
Total	13,616	100.0	
Age group			
18–24	1760	13.7	[13.0–14.5]
25–34	3197	23.6	[22.7–24.5]
35–44	3129	22.6	[21.7–23.5]
45–54	2206	16.4	[15.6–17.2]
55–64	1801	12.9	[12.1–13.6]
65+	1523	10.8	[10.2–11.5]
Total	13,616	100.0	
Education			
Secondary or less	5789	43.0	[41.9–44.1]
Matric	4210	31.9	[30.9–33.0]
Tertiary	3519	25.1	[24.2–26.0]
Total	13,518	100.0	
Income			
R800 or less	1570	16.2	[15.3–17.2]
R801–R3200	3525	37.2	[35.9–38.4]
R3201–R12800	2684	27.9	[26.8–29.2]
R12801 or more	1921	18.7	[17.7–19.7]
Total	9700	100.0	
Medical aid			
Yes	3356	23.2	[22.3–24.1]
No	10,206	76.8	[75.9–77.7]
Total	13,562	100.0	
Living standard satisfaction			
Satisfied	7963	58.3	[57.3–59.4]
Neither satisfied nor dissatisfied	1467	11.4	[10.7–12.1]
Dissatisfied	4186	30.2	[29.2–31.2]
Total	13,616	100.0	
Food satisfaction			
Satisfied	10,923	79.0	[78.1–79.9]
Neither satisfied nor dissatisfied	785	6.4	[5.8–7.0]
Dissatisfied	1908	14.6	[13.8–15.4]
Total	13,616	100.0	
Food expenditure			
R1000 or less	4559	34.8	[33.7–35.9]
R1001–R2000	4400	35.7	[34.6–36.8]
R2001 or more	3807	29.5	[28.5–30.5]
Total	12,766	100.0	
Health status			
Excellent	4446	33.2	[32.2–34.3]
Good	7431	54.4	[53.3–55.5]
Poor	1739	12.4	[11.6–13.1]
Total	13,616	100.0	
Hypertension			
No	10,338	76.8	[75.8–77.7]
Yes	3278	23.2	[22.3–24.2]
Total	13,616	100.0	
Healthcare facilities access			
No	3389	25.7	[24.8–26.7]
Yes	9672	74.3	[73.3–75.2]
Total	13,061	100.0	
Healthcare facility type			
Private	3569	25.3	[24.4–26.3]
Public	9377	74.7	[73.7–75.6]
Total	12,946	100.0	
Health services satisfaction			
Satisfied	8868	65.8	[64.8–66.9]
Neither satisfied nor dissatisfied	994	8.0	[7.4–8.6]
Dissatisfied	3199	26.2	[25.2–27.2]
Total	13,061	100.0	
Dwelling Type			
Formal	11,541	85.6	[84.8–86.3]
Informal/Other	2075	14.4	[13.7–15.2]
Total	13,616	100.0	
District			
City of Ekurhuleni	2963	25.4	[24.5–26.3]
City of Johannesburg	3545	38.4	[37.3–39.5]
City of Tshwane	2810	24.1	[23.1–25.0]
Sedibeng	2160	6.1	[5.7–6.4]
West Rand	2138	6.1	[5.8–6.4]
Total	13,616	100.0	

CI = confidence interval. Subtotals are not always equal to the overall total due to non-response or missing data.

**Table 2:** Prevalence of household self-reported diabetes mellitus in Gauteng, South Africa.

	Sample	%	95% CI	*p* Value
Total	13,616	11.1	[10.4–11.9]	
Sex				
Male	6340	10.5	[9.5–11.7]	0.135
Female	7276	11.7	[10.8–12.6]	
Population group				
Black African	10,933	9.1	[8.5–9.8]	<0.001
Other	2683	19.2	[16.9–21.6]	
Age group				
18–24	1760	6.0	[4.9–7.3]	<0.001
25–34	3197	6.9	[5.8–8.1]	
35–44	3129	6.7	[5.7–7.9]	
45–54	2206	12.5	[10.3–15.0]	
55–64	1801	19.8	[17.5–22.4]	
65+	1523	23.6	[20.8–26.7]	
Education				
Secondary or less	5789	11.5	[10.4–12.6]	0.502
Matric	4210	10.5	[9.1–12.0]	
Tertiary	3519	11.3	[10.0–12.7]	
Income				
R800 or less	1570	7.3	[5.8–9.3]	0.001
R801–R3200	3525	11.0	[9.7–12.4]	
R3201–R12800	2684	11.7	[10.1–13.5]	
R12801 or more	1921	12.9	[10.9–15.3]	
Medical aid				
Yes	3356	13.7	[12.2–15.4]	<0.001
No	10,206	10.3	[9.5–11.1]	
Living standard satisfaction				
Satisfied	7963	12.5	[11.5–13.6]	<0.001
Neither satisfied nor dissatisfied	1467	11.3	[9.4–13.4]	
Dissatisfied	4186	8.3	[7.3–9.4]	
Food satisfaction				
Satisfied	10,923	11.3	[10.6–12.2]	0.184
Neither satisfied nor dissatisfied	785	12.6	[8.7–17.9]	
Dissatisfied	1908	9.2	[7.7–11.0]	
Food expenditure				
R1000 or less	4559	8.3	[7.4–9.4]	<0.001
R1001–R2000	4400	11.6	[10.4–12.9]	
R2001 or more	3807	13.9	[12.3–15.7]	
Health status				
Excellent	4446	7.1	[6.2–8.1]	<0.001
Good	7431	10.9	[9.9–12.0]	
Poor	1739	22.7	[20.0–25.7]	
Hypertension				
No	10,338	6.1	[5.4–6.8]	<0.001
Yes	3278	27.7	[25.7–29.8]	
Healthcare facilities access				
No	3389	12.0	[10.6–13.5]	0.281
Yes	9672	11.1	[10.2–12.0]	
Healthcare facility type				
Private	3569	13.6	[12.2–15.3]	<0.001
Public	9377	10.6	[9.7–11.5]	
Health services satisfaction				
Satisfied	8868	11.6	[10.7–12.7]	0.380
Neither satisfied nor dissatisfied	994	10.1	[8.0–12.7]	
Dissatisfied	3199	10.8	[9.5–12.2]	
Dwelling type				
Formal	11,541	12.2	[11.4–13.0]	<0.001
Informal/Other	2075	4.7	[3.4–6.4]	

CI = confidence interval. Subtotals are not always equal to the overall total due to non-response or missing data.

**Table 3:** Multivariate logistic regression model of factors associated with household self-reported diabetes mellitus.

	aOR	[95% CI]	*p* Value
Population group			
Black African	1.00		
Other	1.70	[1.33–2.18]	<0.001
Age group			
18–24	1.00		
25–34	1.28	0.95–1.73	0.109
35–44	1.03	0.76–1.40	0.831
45–54	1.74	1.25–2.42	0.001
55–64	2.38	1.77–3.20	<0.001
65+	2.16	1.57–2.96	<0.001
Medical aid			
Yes	1.00		
No	1.12	0.87–1.43	0.386
Living standard satisfaction			
Satisfied	1.00		
Neither satisfied nor dissatisfied	1.00	0.78–1.30	0.97
Dissatisfied	0.88	0.71–1.08	0.224
Food satisfaction			
Satisfied	1.00		
Neither satisfied nor dissatisfied	1.58	0.97–2.58	0.065
Dissatisfied	1.03	0.80–1.34	0.806
Food expenditure			
R1000 or less	1.00		
R1001–R2000	1.44	1.18–1.74	<0.001
R2001 or more	1.41	1.11–1.80	0.006
Health status			
Excellent	1.00		
Good	1.19	0.98–1.44	0.084
Poor	2.13	1.65–2.75	<0.001
Hypertension			
No	1.00		
Yes	4.26	3.58–5.08	<0.001
Dwelling type			
Formal	1.00		
Informal/Other	0.51	0.37–0.69	<0.001

CI = confidence interval. aOR = adjusted odds ratio.

## Data Availability

Data are available from https://www.datafirst.uct.ac.za/dataportal/index.php/collections/GCRO. Accessed on 2 October 2023.

## References

[1] Baynes H.W. (2015). Classification, pathophysiology, diagnosis and management of diabetes mellitus. J. Diabetes Metab..

[2] Bettencourt-Silva R., Aguiar B., Sá-Araújo V., Barreira R., Guedes V., Ribeiro M.J.M., Carvalho D., Östlundh L., Paulo M.S. (2019). Diabetes-related symptoms, acute complications and management of diabetes mellitus of patients who are receiving palliative care: A protocol for a systematic review. BMJ Open.

[3] Ong K.L., Stafford L.K., Mclaughlin S.A., Boyko E.J., Vollset S.E., Smith A.E., Dalton B.E., Duprey J., Cruz J.A., Hagins H. (2023). Global, regional, and national burden of diabetes from 1990 to 2021, with projections of prevalence to 2050: A systematic analysis for the Global Burden of Disease Study 2021. Lancet.

[4] Ohiagu F.O., Chikezie P.C., Chikezie C.M. (2021). Pathophysiology of diabetes mellitus complications: Metabolic events and control. Biomed. Res. Ther..

[5] International Diabetes Federation (2021). IDF Diabetes Atlas.

[6] Sifunda S., Mbewu A.D., Mabaso M., Manyaapelo T., Sewpaul R., Morgan J.W., Harriman N.W., Williams D.R., Reddy S.P. (2023). Prevalence and Psychosocial Correlates of Diabetes Mellitus in South Africa: Results from the South African National Health and Nutrition Examination Survey (SANHANES-1). Int. J. Environ. Res. Public Health.

[7] Stokes A., Berry K.M., Mchiza Z., Parker W., Labadarios D., Chola L., Hongoro C., Zuma K., Brennan A.T., Rockers P.C. (2017). Prevalence and unmet need for diabetes care across the care continuum in a national sample of South African adults: Evidence from the SANHANES-1, 2011–2012. PLoS ONE.

[8] Grundlingh N., Zewotir T.T., Roberts D.J., Manda S. (2022). Assessment of prevalence and risk factors of diabetes and pre-diabetes in South Africa. J. Health Popul. Nutr..

[9] Stats SA (2017). Mortality and Causes of Death in South. Africa, 2016: Findings from Death Notification.

[10] Galicia-Garcia U., Benito-Vicente A., Jebari S., Larrea-Sebal A., Siddiqi H., Uribe K.B., Ostolaza H., Martín C. (2020). Pathophysiology of type 2 diabetes mellitus. Int. J. Mol. Sci..

[11] Boden-Albala B., Elkind M.S., White H., Szumski A., Paik M.C., Sacco R.L. (2009). Dietary total fat intake and ischemic stroke risk: The northern Manhattan study. Neuroepidemiol.

[12] Azétsop J., Joy T.R. (2013). Access to nutritious food, socioeconomic individualism and public health ethics in the USA: A common good approach. Philos. Ethics Humanit. Med..

[13] Mokhele S., Aboyade O., Katerere D.R. (2024). Obesity Prevention Effects of Avocado (Persea americana) Seed Powder in High-Fat Diet-Induced Obesity in Rats. Nutraceuticals.

[14] Pheiffer C., Pillay-Van Wyk V., Turawa E., Levitt N., Kengne A.P., Bradshaw D. (2021). Prevalence of type 2 diabetes in South Africa: A systematic review and meta-analysis. Int. J. Environ. Res. Public Health.

[15] Ruze R., Liu T., Zou X., Song J., Chen Y., Xu R., Yin X., Xu Q. (2023). Obesity and type 2 diabetes mellitus: Connections in epidemiology, pathogenesis, and treatments. Front. Endocrinol..

[16] Klobučar S., Detel D., Igrec M., Bergoč A., Rahelić V., Rahelić D. (2024). Overweight and obesity in adults with Type 1 Diabetes: A growing challenge. Diabetology.

[17] Shisana O., Labadarios D., Rehle T., Simbayi L., Zuma K., Dhansay A., Reddy P., Parker W., Hoosain E., Naidoo P. (2013). The South African National Health and Nutrition Examination Survey (SANHANES-1).

[18] Mokhele S., Tswaledi D., Aboyade O., Shai J., Katerere D.R. (2020). Investigation of Aloe ferox leaf powder on anti-diabesity activity. S. Afr. J. Bot..

[19] Mutyambizi C., Booysen F., Stokes A., Pavlova M., Groot W. (2019). Lifestyle and socioeconomic inequalities in diabetes prevalence in South Africa: A decomposition analysis. PLoS ONE.

[20] Magodoro I.M., Okello S., Dungeni M., Castle A.C., Mureyani S., Danaei G. (2022). Association between HIV and prevalent hypertension and diabetes mellitus in South Africa: Analysis of a nationally representative cross-sectional survey. Int. J. Infect. Dis..

[21] Stats SA (2023). Census 2022: Provinces at a Glance.

[22] NDoH, Stats SA, SAMRC, ICF (2019). South Africa Demographic and Health Survey 2016.

[23] Okura Y., Urban L.H., Mahoney D.W., Jacobsen S.J., Rodeheffer R.J. (2004). Agreement between self-report questionnaires and medical record data was substantial for diabetes, hypertension, myocardial infarction and stroke but not for heart failure. J. Clin. Epidemiol..

[24] Moradinazara M., Pasdarb Y., Najafia F., Shakibac E., Hamzehd B., Samadib M., Mirzaeia M., Dobson A.J. (2020). Validity of self-reported diabetes varies with sociodemographic charecteristics: Example from Iran. Clin. Epidemiol. Glob. Health.

[25] Ho S.Y., Mak K.K., Thomas G.N., Schooling M., Fielding R., Janus E.D., Lam T.H. (2007). The relation of chronic cardiovascular diseases and diabetes mellitus to perceived health, and the moderating effects of sex and age. Soc. Sci. Med..

[26] Neethling A. (2021). Quality of Life Survey 6 (2020/21): Weighting Report.

[27] Hamann C., de Kadt J. (2021). GCRO Quality of Life Survey 6 (2020/21): Sample Design.

[28] Moeti T., Mokhele T., Weir-Smith G., Dlamini S., Tesfamichael S. (2023). Factors affecting access to public healthcare facilities in the City of Tshwane, South Africa. Int. J. Environ. Res. Public Health.

[29] (2017). Stata Corp.

[30] Kalyani R.R., Golden S.H., Cefalu W.T. (2017). Diabetes and aging: Unique considerations and goals of care. Diabetes Care.

[31] Tsimihodimos V., Gonzalez-Villalpando C., Meigs J.B., Ferrannini E. (2018). Hypertension and diabetes mellitus: Coprediction and time trajectories. Hypertension.

[32] Kristo A.S., Izler K., Grosskopf L., Kerns J.J., Sikalidis A.K. (2024). Emotional eating is associated with T2DM in an urban Turkish population: A pilot study utilizing social media. Diabetology.

[33] Zwane J., Modjadji P., Madiba S., Moropeng L., Mokgalaboni K., Mphekgwana P.M., Kengne A.P., Mchiza Z.J.-R. (2023). Self-management of diabetes and associated factors among patients seeking chronic care in Tshwane, South Africa: A facility-based study. Int. J. Environ. Res. Public Health.

[34] Manne-Goehler J., Atun R., Stokes A., Goehler A., Houinato D., Houehanou C., Hambou M.M.S., Mbenza B.L., Sobngwi E., Balde N. (2016). Diabetes diagnosis and care in sub-Saharan Africa: Pooled analysis of individual data from 12 countries. Lancet Diabetes Endocrinol..

[35] Ataguba J.E., Akazili J., McIntyre D. (2011). Socioeconomic-related health inequality in South Africa: Evidence from General Household Surveys. Int. J. Equity Health.

[36] Madela S.L.M., Harriman N.W., Sewpaul R., Mbewu A.D., Williams D.R., Sifunda S., Manyaapelo T., Nyembezi A., Reddy S.P. (2023). Area-level deprivation and individual-level socioeconomic correlates of the diabetes care cascade among Black South Africans in uMgungundlovu, KwaZulu-Natal, South Africa. PLoS ONE.

[37] World Health Organisation (1996). Health Interview Surveys: Towards International Harmonization of Methods and Instruments.

[38] Miilunpalo S., Vuori I., Oja P., Pasanen M., Urponen Copenhagen H. (1997). Self-rated health status as a health measure: The predictive value of self-reported health status on the use of physician services and on mortality in the working-age population. J. Clin. Epidemiol..

[39] Wu S., Wang R., Zhao Y., Ma X., Wu M., Yan X., He J. (2013). The relationship between self-rated health and objective health status: A population-based study. BMC Public Health.

[40] Mlangeni L., Mabaso M., Makola L., Zuma K. (2019). Predictors of poor self-rated health in KwaZulu-Natal, South Africa: Insights from a cross-sectional survey. Open Public Health J..

[41] Kasenda S., Meland E., Hetlevik Ø., Thomas Mildestvedt T., Dullie L. (2022). Factors associated with self-rated health in primary care in the South-Western health zone of Malawi. BMC Prim. Care.

[42] Mokhele T., Mutyambizi C., Manyaapelo T., Ngobeni A., Ndinda C., Hongoro C. (2023). Determinants of deteriorated self-perceived health status among informal settlement dwellers in South Africa. Int. J. Environ. Res. Public Health.

